# Comparative Analysis of Hematological and Biochemical Changes in Neonates among Women with and without COVID-19 Infection during Pregnancy

**DOI:** 10.3390/children10081370

**Published:** 2023-08-10

**Authors:** Daniela-Eugenia Popescu, Simona Cerbu, Ioana Rosca, Nicoleta Lungu, Ana Adriana Trușculescu, Valerica Belengeanu, Aniko Maria Manea, Mirabela Adina Dima, Florin Gorun, Zoran Laurentiu Popa, Doru Ciprian Crisan, Marioara Boia

**Affiliations:** 1 Department of Obstetrics-Gynecology and Neonatology, “Victor Babes” University of Medicine and Pharmacy, 300041 Timisoara, Romania; popescu.daniela@umft.ro (D.-E.P.); lungu.nicoleta@umft.ro (N.L.); manea.aniko@umft.ro (A.M.M.); dima_mirabela@yahoo.com (M.A.D.); zoranpopal@yahoo.com (Z.L.P.); crisan.doru@umft.ro (D.C.C.); boia.marioara@umft.ro (M.B.); 2 Department of Neonatology, Premiere Hospital, Regina Maria Health Network, 300645 Timisoara, Romania; 3 Discipline of Radiology and Medical Imaging, “Victor Babes” University of Medicine and Pharmacy Timisoara, Eftimie Murgu Square No. 2, 300041 Timisoara, Romania; 4 Neonatology Department, Clinical Hospital of Obstetrics and Gynecology “Prof. Dr. P.Sirbu”, 060251 Bucharest, Romania; ioana.rosca@umfcd.ro; 5 Faculty of Midwifery and Nursery, University of Medicine and Pharmacy “Carol Davila”, 020021 Bucharest, Romania; 6 Department of Neonatology, “Louis Țurcanu” Children Emergency Clinical Hospital Timișoara, 300011 Timisoara, Romania; 7 Pulmonology Department, “Victor Babes” University of Medicine and Pharmacy, Eftimie Murgu Square 2, 300041 Timisoara, Romania; ana.trusculescu@umft.ro; 8 Center for Research and Innovation in Precision Medicine of Respiratory Diseases (CRIPMRD), “Victor Babes” University of Medicine and Pharmacy, 300041 Timisoara, Romania; 9 Department of Genetics, Institute of Life Science, Faculty of Medicine, “Vasile Goldiş” Western University of Arad, 310025 Arad, Romania; belengeanu.valerica@student.uvvg.ro; 10 Department of Obstetrics and Gynecology, Municipal Emergency Clinical Hospital Timisoara, 300172 Timisoara, Romania; gorun.florin@umft.ro

**Keywords:** COVID-19, vertical transmission, pregnancy complications

## Abstract

The aim of this study is to evaluate the test results of neonates delivered by COVID-19-positive mothers during pregnancy with those of neonates born to unvaccinated mothers who are COVID-19-free. A cohort study was conducted on 367 pregnant women who gave birth at Premiere Hospital, Timisoara, Romania, between May 2021 and February 2022. Two groups were established: Group 1, with 167 pregnant women infected with COVID-19, and Group 2, with 200 pregnant women who were not affected by COVID-19 during pregnancy. Maternal laboratory examination did not exhibit significant variations except for platelet count. In neonatal blood tests, WBC had a significantly lower median value in the group born to COVID-19-free mothers. Neonatal anemia and leukocytosis showed slightly higher prevalence in Group 1, but the differences were not statistically significant. This study suggests that maternal COVID-19 infection during pregnancy does not have significant associations with most maternal and neonatal characteristics.

## 1. Introduction

The COVID-19 (Appendix A) pandemic has exerted a profound impact on global health, exerting its influence upon individuals spanning diverse age groups and populations [1,2]. Of particular concern was the potential transmission of SARS-CoV-2 from infected women during pregnancy to their developing fetuses [3,4]. According to current data, maternal infection with COVID-19 during pregnancy could have consequences for the outcome of newborns. Recent investigations have revealed an increased risk of adverse newborn outcomes, including preterm birth, respiratory distress, and admission to the neonatal intensive care unit (NICU), among newborns born to COVID-19-affected mothers [5,6,7,8]. Several recent studies have evaluated the rate of fetal complications in neonates born to COVID-19-positive mothers. In a study of 503 newborns born to COVID-19-positive mothers, the rate of prematurity was reported to be 15.7%, and the rate of very premature births was 5.2% [9]. However, a systematic review of the literature that includes case reports and case series estimates preterm birth at 25.9%. Furthermore, the rate of low birth weight for gestational age was estimated at 8.3%, low birth weight at 15.6%, birth asphyxia at 1.8%, and respiratory distress syndrome had a rate of 6.4% [10]. Nevertheless, pregnancy outcomes in COVID-19-positive women also seem to be influenced by the SARS-CoV-2 variant. Preterm birth below 37 gestational weeks (GW) among COVID-19-positive women was estimated at 17.5% in the pre-Delta wave and 25.2% in the Delta wave. Significant differences were observed, however, in the preterm birth rate below 34 GW, 4.9% pre-Delta, and 15.4% during the Delta wave. The Omicron variant appears to be less associated with preterm birth during the wave of this variant, with the birth rate under 37 weeks estimated at 8.3% and under 34 weeks at 2.8% [11]. Additionally, neonates delivered to mothers with COVID-19 from disadvantaged socioeconomic backgrounds were more likely to have complications, such as premature birth [12].

Pathological evidence has shown that COVID-19-affected pregnancies may have vascular malperfusion and fetal vessel thrombosis in the placenta. These changes occur even in the absence of inflammatory changes or confirmed placental infection. It is possible that these placental changes are a result of a maternal systemic hypercoagulable or hyperinflammatory state following COVID-19 infection [13,14,15]. Platelets are crucial factors in promoting inflammation, and their role in viral infection-mediated thrombosis is well known [16,17]. SARS-CoV-2 viral particles enter the maternal circulation during COVID-19 infection. The virus triggers a robust immune response characterized by increased production of cytokines, such as interleukin-6 (IL-6) and interleukin-1β (IL-1β). The elevated cytokines lead to endothelial cell activation and dysfunction, resulting in increased expression of adhesion molecules and tissue factors [18]. This process promotes platelet adhesion and aggregation at the site of inflammation. Activated endothelial cells release von Willebrand factor (vWF) and other pro-thrombotic factors, further promoting platelet activation and recruitment [19]. In response to the inflammatory milieu, megakaryocytes in the bone marrow increase platelet production and release, contributing to elevated platelet levels [20]. Pregnancy itself induces physiological changes, such as hormonal fluctuations and altered hemodynamics, which can influence platelet function and count. The combination of COVID-19 and pregnancy might exacerbate these effects, leading to a more pronounced elevation in platelet levels.

Nonetheless, a comprehensive grasp of the hematological and biochemical profiles of these neonates remains somewhat constrained.

Hematological and biochemical analyses are indispensable diagnostic instruments that furnish significant insights into the overall well-being and physiological condition of neonates. These analyses encompass a wide array of indicators comprised of comprehensive blood counts, liver function evaluations, and renal function assessments [21,22].

The aim of this retrospective analysis is to compare the hematological and biochemical test results of infants born to mothers who were discovered to be positive for COVID-19 during the gestational period with those born to unvaccinated mothers who were found to be free of COVID-19 during pregnancy.

## 2. Materials and Methods

### 2.1. Study Design and Settings

A retrospective cohort study was conducted on 367 infants born at Première Hospital (Regina Maria Healthcare Network), Timisoara, Romania, between May 2021 and February 2022. The participants were divided into two groups: Group 1 contained 167 pregnant women who had been infected with COVID-19 while pregnant, and Group 2 consisted of 200 pregnant women who had not been affected by COVID-19 during the same gestational period. The research methodology employed in this study has received approval from the Ethics Commission Board at Première Hospital (No. 330/18.11.2021), as well as the Ethics Committee of Scientific Research at “Victor Babeș” University of Medicine and Pharmacy Timișoara (No. 76/2020). The study design adhered scrupulously to the principles encapsulated in the Declaration of Helsinki.

### 2.2. Participants

The criteria for selection of participants were comprised of women who gave birth to live-born infants at Première Hospital- Regina Maria Healthcare Network, Timisoara, within the timeframe of May 2021 and February 2022. The age of the women included in the study ranged from 21 to 47 years old.

The investigation excluded those participants who had incomplete or inadequate information in their medical records, which had the potential to impact the analysis or interpretation. The research also excluded pregnant women who had received the COVID-19 vaccine in order to focus on comparing neonates born to mothers with natural COVID-19 infection with those born to unvaccinated mothers who did not have COVID-19 during pregnancy. The exclusion of vaccinated patients facilitated the isolation of the effects of maternal COVID-19 infection.

### 2.3. Variables

The existing investigation comprised an examination of neonatal hematological parameters, specifically the measurement of white blood cells, red blood cells, hemoglobin, hematocrit, and platelets, together with neonatal biochemical parameters, particularly assessments of liver function (such as alanine aminotransferase and aspartate aminotransferase) and renal function (such as serum creatinine and blood urea nitrogen).

The exposure of interest of our study pertained to the infection of COVID-19 in expectant mothers during gestation.

To explore potential predictors, we also considered maternal age, as it may influence neonatal outcomes and laboratory parameters. We assessed the severity of maternal COVID-19 symptoms as an additional predictor, recognizing its potential impact on neonatal outcomes and hematological/biochemical profiles. Furthermore, the mode of delivery (vaginal or cesarean section) was considered as a predictor, considering its influence on neonatal outcomes and laboratory parameters.

In this study, we did not identify any specified effect modifiers. However, our analysis was based on comprehensive data collected from the medical records of 367 participants. To enhance the credibility and reliability of our findings, we ensured the inclusion of complete data and excluded vaccinated patients.

To diagnose maternal COVID-19 infection, we relied on laboratory-confirmed diagnostic tests, such as reverse transcription-polymerase chain reaction (RT-PCR). The presence or absence of COVID-19 infection during pregnancy was documented in the medical records of the participants.

### 2.4. Data Sources/Measurement

Data for variables in this study were obtained from complete medical records available for each participant.

To assess the neonatal hematological parameters, blood samples were collected shortly after birth, and laboratory tests were conducted.

### 2.5. Reducing Bias

In order to abate plausible origins of bias in the current investigation, a variety of techniques were implemented. The present analysis utilized a retrospective study design and collected medical records of all patients who gave birth during the study period. The primary goal of this design was to mitigate potential selection bias. Furthermore, the carefully crafted eligibility criteria entail a crucial requirement of possessing comprehensive and dependable medical records. This particular criterion serves as a crucial measure to mitigate the potential occurrence of bias stemming from missing or incomplete data. In addition, individuals who received the COVID-19 vaccine during pregnancy were excluded from this study. By excluding vaccinated individuals, the potential bias introduced by the effects of vaccination on neonatal outcomes and laboratory parameters was mitigated. Finally, the statistical models were designed to incorporate pertinent covariates, such as maternal age, comorbidities, and mode of birth, in an effort to effectively manage their impact on neonatal outcomes and laboratory parameters.

### 2.6. Statistical Analysis

IBM SPSS v.29.0 (IBM Corp., Armonk, NY, USA: IBM Corp., Armonk, NY, USA) was used to conduct all analyses. Independent *t*-tests or Mann–Whitney U tests were used to compare continuous variables, such as hematological and biochemical parameters, between the COVID-positive and COVID-negative groups. To assess associations between categorical variables, such as neonatal outcomes and maternal COVID-19 infection status, chi-square tests or Fisher’s exact tests were used.

## 3. Results

### 3.1. Baseline Characteristics

There were no significant differences between Group 1 (COVID-19-positive) and Group 2 (free-COVID-19) in terms of maternal characteristics such as age, gestation, parity, diabetes, hypertension, cardiovascular disease, and hepatitis. Additionally, there were no noteworthy distinctions between the two groups with regard to pregnancy complications such as C-sections, preeclampsia, fetal growth restriction (FGR), and preterm birth. However, there was a statistically significant difference in the gender distribution between the groups, with Group 2 having a slightly higher proportion of male infants. Furthermore, there were no significant differences between the groups in terms of gestational age at birth, weight, length, head circumference, and APGAR scores (Table 1). The infants’ birth weight ranged from 1800 g to 4960 g, with a higher median value in Group 1, but this difference was not statistically significant (*p* = 0.07) (Table 1).

### 3.2. Investigating the Impact of Maternal COVID-19 Infection on Fetal Laboratory Test Results

In regard to the maternal laboratory examination conducted at the moment of delivery, the findings did not exhibit any statistically noteworthy variations between the two cohorts for the majority of the measurements, except for the platelet count (Table 2).

On the other hand, in neonatal blood tests, WBC had a statistically significantly lower median value in the group of those born to COVID-19-free mothers during pregnancy (Table 2).

In bold are the parameters showing a statistical difference between groups. Moreover, the analysis presented in Table 3 examines potential associations between the COVID-19 condition during pregnancy and the occurrence of specific laboratory abnormalities in newborns. Neonatal anemia was present in 11.4% of cases in Group 1 compared to 7.5% in Group 2, but the difference was not statistically significant (*p* = 0.21). Similarly, neonatal leukocytosis showed a slightly higher prevalence in Group 1 (5.39%) compared to Group 2 (3.0%), but the difference was not statistically significant (*p* = 0.29) (Table 3).

Regarding other parameters, the study found that neonatal thrombocytopenia, elevated GOT levels, elevated GPT levels, elevated conjugated hyperbilirubinemia, and elevated creatinine levels had very low occurrence rates or were not present in Group 2. Therefore, statistical analyses such as *p*-values and odds ratios were not applicable for those specific parameters.

### 3.3. Hematological and Biochemical Profiles in Neonates of COVID-19-Positive Mothers

A subgroup analysis was conducted to specifically examine the hematological and biochemical profiles of neonates born to mothers who tested positive for COVID-19 during pregnancy. Among the 167 participants, 13.77% were asymptomatic, while the majority experienced respiratory symptoms (65.87%). Other commonly reported symptoms included fever (28.74%), anosmia (39.52%), ageusia (33.53%), headache (19.76%), and diarrhea (1.80%). Additionally, 5.39% of the individuals required hospitalization due to COVID-19.

**Figure 1:**
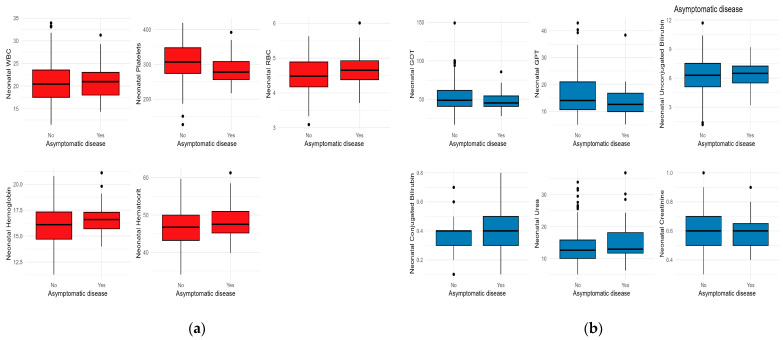
Comparative analysis of neonatal laboratory test medians according to the presence of COVID-19 symptoms: (**a**) Hematological tests; (**b**) Biochemical laboratory tests. Note: Black dots represent outliers; The whiskers extend from the minimum to the lower quartile and from the upper quartile to the maximum; Red colour represents hematological tests and and blue colour biochemical blood tests.

Figure 1 presents the hematological values of newborns whose mothers were infected with COVID-19 during pregnancy, comparing those born to mothers with symptomatic COVID-19 during pregnancy and those born to mothers with asymptomatic COVID-19 during pregnancy. The results indicate that there were no significant differences in white blood cell (WBC) counts (*p* = 0.64) and hemoglobin levels (*p* = 0.33) between newborns of symptomatic and asymptomatic mothers. However, there was a significant difference in platelet counts (*p* = 0.04), with newborns of symptomatic mothers having a higher median count of 307 (with an interquartile range of 73.75) compared to 278 (with an interquartile range of 51.50) in newborns of asymptomatic mothers. No significant differences were observed in hematocrit levels (*p* = 0.35) and red blood cell (RBC) count (*p* = 0.18) between the two groups.

No significant differences were found in liver function (GOT, GPT), bilirubin levels (unconjugated, conjugated), or renal function (urea, creatinine) between newborns of symptomatic and asymptomatic mothers (Figure 1).

**Figure 2:**
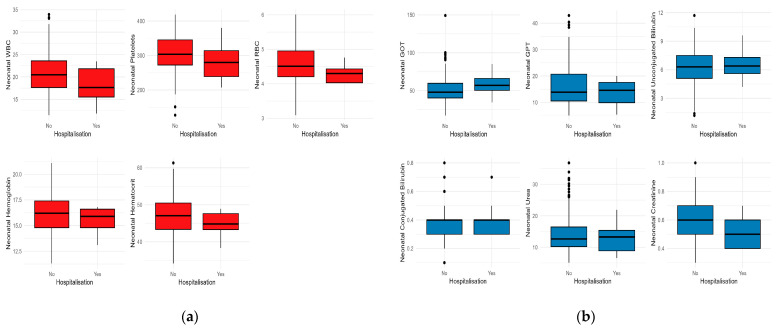
Comparative analysis of neonatal laboratory test medians according to the presence of COVID-19 hospitalization: (**a**) Hematological tests; (**b**) Biochemical laboratory tests. Note: Black dots represent outliers; The whiskers extend from the minimum to the lower quartile and from the upper quartile to the maximum; Red colour represents hematological tests and and blue colour biochemical blood tests.

Moreover, depending on the necessity of hospitalization due to COVID-19, a lower median value of hemoglobin and hematocrit of newborns is observed among those born to women hospitalized for COVID-19. In contrast, neonatal GOT and GPT levels were slightly higher among those born to women who required hospitalization for COVID-19 (Figure 2).

**Figure 3:**
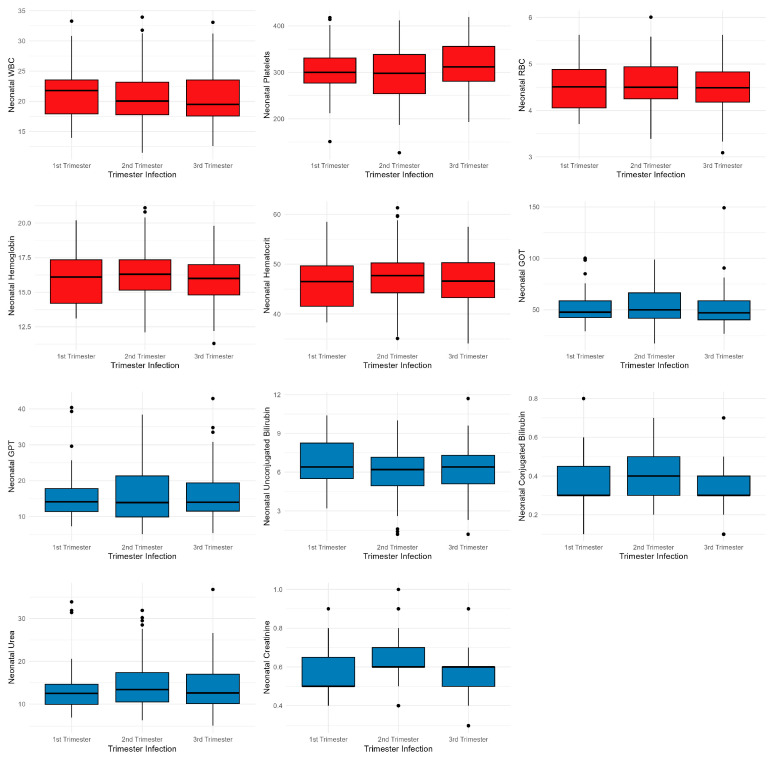
Comparative analysis of neonatal laboratory test medians according to the trimester of maternal infection with COVID-19. Note: Black dots represent outliers; The whiskers extend from the minimum to the lower quartile and from the upper quartile to the maximum; Red colour represents hematological tests and and blue colour biochemical blood tests.

In addition, analysis of neonatal laboratory test results showed no significant differences according to the trimester of maternal infection with COVID-19 (Figure 3.)

**Figure 4:**
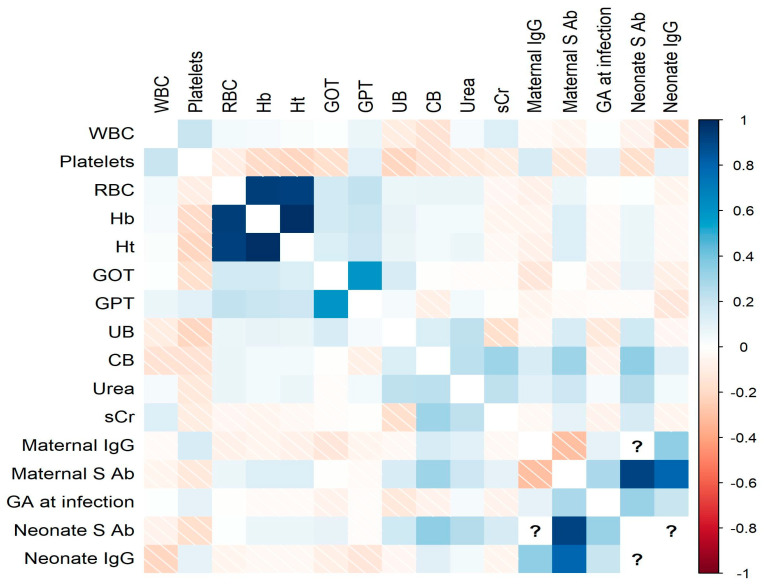
Correlation matrix plot presenting pairwise correlation coefficients between neonatal hematological, biochemical, and immunological findings. Note: Sign “?” = “not applicable”; as none of the participants were tested for both IgG and anti-S antibodies.

The correlation analysis investigated the relationships between various laboratory parameters in both maternal and neonatal samples. Among the maternal parameters, there were several noteworthy correlations. Maternal IgG levels showed a positive correlation with neonatal IgG levels, suggesting the potential transfer of maternal antibodies to the newborn. Additionally, maternal spike antibody levels were positively correlated with neonatal spike antibody levels (Figure 4).

In terms of neonatal laboratory outcomes, depending on the presence of maternal COVID-19 symptoms, neonatal anemia was detected only in neonates that were born to symptomatic women. Additionally, neonatal leukocytosis was more frequent among them but with a statistically insignificant value (Table 4). In addition, neonatal anemia was more common in infants born to mothers who required hospitalization due to COVID-19, although the difference was statistically insignificant.

Additionally, neonatal laboratory outcomes revealed no statistically significant differences according to maternal or neonatal COVID-19 immunopositivity (Table 5).

## 4. Discussion

### 4.1. Key Results

The analysis compared two groups of expecting pregnant women: Group 1 was comprised of mothers who tested positive for COVID-19 during pregnancy, while Group 2 was comprised of COVID-19-free pregnant women. The baseline characteristics of the women and neonates were not significantly different between the two groups. In terms of laboratory test results, there were no statistically significant differences between the two groups, except for the platelet count, which was higher in women who had COVID-19 during pregnancy. In terms of neonatal blood tests, white blood cell counts showed a statistically significant lower median value among newborns born to mothers who did not have COVID-19. The prevalence of neonatal anemia and neonatal leukocytosis showed a slight increase in Group 1, but this was not statistically significant.

In terms of neonatal laboratory parameters, some interesting associations were observed. Neonatal IgG and Spike antibody levels exhibited a strong positive correlation with maternal IgG and, respectively, spike antibody levels, implying the transfer of antibodies from the mother to the infant during pregnancy.

### 4.2. Limitations

This study has several limitations. First, an observational design was followed, which may limit the ability to establish causality between maternal COVID-19 infection and neonatal hematologic and biochemical outcomes. Second, the sample size was relatively small, with the study being conducted in a single clinic, which may affect the statistical power to detect significant differences. There may also be other unmeasured confounders that could influence the results. In addition, the study only included certain laboratory parameters and did not explore other potential indicators of neonatal health. Furthermore, the study was conducted between May 2021 and February 2022 (wave 3 and wave 4 of the pandemic in Romania), and the findings primarily pertain to the prevalent SARS-CoV-2 variant during that time. As the virus continues to evolve, new variants may emerge with different characteristics and potential impacts on neonatal health. Therefore, the results may not fully capture the effects of more recent or future variants of SARS-CoV-2. The study period coincided with waves 3 and 4 of COVID-19 infections. Temporal changes in cases and healthcare practices during these waves may have influenced neonatal outcomes. However, the study did not directly explore the impact of specific waves on neonatal health. Ultimately, the investigation was focused on a specific demographic, and the transferability of the results to additional demographics or contexts may be limited.

### 4.3. Interpretation

The findings that the neonatal hematologic and biochemical test results, including white blood cell count (WBC), platelets, hemoglobin, hematocrit, red blood cells, glutamic-oxaloacetic transaminase (GOT), and glutamic-pyruvic transaminase (GPT), do not show significant variations related to COVID-19 status during pregnancy. Despite the findings of this investigation, an increasing amount of scientific evidence has shown abnormalities in traditional laboratory analyses, particularly in hematology assays, which may have the potential to predict the disease and improve its clinical monitoring [23].

Our study shows that newborns born to COVID-19-positive mothers during pregnancy have significantly increased levels of white blood cells. This is consistent with literature data which shows that abnormal neonatal WBC is the most common laboratory finding of infants whose mothers had COVID-19 [24]. Furthermore, Al-Matary et al. show in a study of 228 women with COVID-19 during pregnancy that 52.5% of the newborns had WBCs above normal levels and none with leukopenia [25]. However, Kosmeri et al. conclude that leukopenia is the most common WBC abnormality in children with COVID-19, not in infants and neonates, where lymphocytosis was more common [26].

Hepatic injury in both adult and pediatric individuals affected by COVID-19 has been documented in the literature [27,28,29,30]. In line, our study shows a higher level of liver transaminases among newborns born to mothers with COVID-19 during pregnancy. In addition, the prevalence of infants with increased GOT is higher in the group of COVID-19-positive mothers. Furthermore, literature data show GOT levels to be significantly higher than normal among newborns born to COVID-positive mothers [24].

Anemia presents itself as a prevalent occurrence amongst individuals diagnosed with COVID-19, with a frequency ranging from 25.6–61% [31,32,33]. It is well-established that the occurrence of anemia in mothers has a noteworthy influence on the outcomes of neonates. Empirical research has discovered that the occurrence of anemia in mothers is connected to adverse neonatal consequences, such as diminished birth weight, height, and head circumference [34]. In addition, some studies have shown an association between maternal hemoglobin levels and fetal hemoglobin levels [35]. On the contrary, in our study, no differences between maternal and neonatal hemoglobin levels were detected between the two groups. However, the prevalence of neonatal anemia is higher in infants whose mothers suffered from COVID-19 during pregnancy, being exclusively present in newborns born to women with symptomatic disease during gestation. Maternal infections with several viruses, including rubella or cytomegalovirus, are associated with hematological abnormalities in the fetus, such as thrombocytopenia and anemia [36,37]. In addition, the association of severe fetal anemia, which can cause hydrops fetalis, with parvovirus infection has been clearly demonstrated [38]. However, there is no evidence of an association of COVID-19 with fetal anemia. In addition, the hemoglobin level of the umbilical cord blood of newborns from COVID-19-positive mothers has been reported to be in the normal range [15]. This suggests that COVID-19 infection during pregnancy does not directly impact fetal hemoglobin levels.

Patients with mild COVID-19 symptoms show a slight increase in their platelet count, while a decrease in platelet count is a distinguishing feature of severe infections [39]. This statement is supported by our study, where platelet levels are statistically significantly higher in women in the COVID-19-positive group, with the majority of participants having a mild form of the disease without the necessity of hospitalization. However, maternal thrombocytopenia in severe cases may be a reliable marker of fetal demise due to placental inflammatory changes [40]. Furthermore, fetal thrombocytopenia can be exclusively caused by placental insufficiency. The pathology abnormalities that comprise SARS-CoV-2 placentitis lead to extensive and severe destruction of the placenta, culminating in placental malperfusion and insufficiency [41]. However, in our study, neonatal thrombocytopenia was found in only one case, where vertical transmission was also evident. In contrast, a systematic review showed that a low platelet count was found in about 29% of cases [24].

COVID-19 vertical transmission is a topic of concern and ongoing research.

Our results showed that maternal spike antibody levels were positively correlated with neonatal spike antibody levels, indicating a possible transmission of spike antibodies from the mother to the baby. This finding underscores the potential role of maternal antibodies in conferring passive immunity to the neonate. Studies in the literature are contradictory regarding vertical transmission. According to the evidence, the possibility of vertical transmission of COVID-19 is rare, but reports suggesting it is increasing [42,43]. However, our evidence does not show a negative association of antibody transmission on fetal hematological and biochemical tests.

## 5. Conclusions

In conclusion, this study indicates that there were no significant differences between COVID-19-positive mothers and mothers without COVID-19 during pregnancy in terms of neonatal hematological and biochemical characteristics. The study provides important preliminary insight into the association between maternal COVID-19 infection during pregnancy and neonatal outcomes, but further research with larger and more diverse populations is needed to validate these findings and increase generalizability. Considering the evolving nature of the SARS-CoV-2 virus and the emergence of new variants, it is essential to continuously update laboratory testing protocols to account for any potential changes in neonatal health outcomes. Based on the study’s findings, we propose routine laboratory testing of certain parameters in neonates born to mothers with COVID-19 during pregnancy. Specifically, we recommend regular monitoring of hematological parameters, including complete blood counts and biochemical markers, such as liver function tests, in neonates born to mothers with confirmed COVID-19 infection. Timely assessment of these parameters can aid in the early detection of any potential complications and enable timely interventions, thereby improving neonatal outcomes.

## Figures and Tables

**Table 1:** Baseline characteristics of mothers and neonates included in the study.

Parameter	Overall	Group 1 (n = 167/45.50%)	Group 2 (n = 200/54.50%)	*p*-Value
*Maternal Characteristics*				
Age	33 (6)	32 (5)	33 (6)	0.06
Gestation	2 (1)	2(1)	1 (1)	0.39
Parity	1 (1)	1 (1)	1 (1)	0.12
Diabetes	2 (0.54%)	0	2	*NA*
Hypertension	2 (0.54%)	0	2	*NA*
Cardiovascular disease	2 (0.54%)	2 (1.19%)	0	*NA*
Hepatitis	6 (1.63%)	2 (1.19%)	4 (2.0%)	0.84
*Trimester of infection*				
1st trimester	-	37 (22.3%)		
2nd trimester	-	73 (44%)		
3rd trimester	-	56 (33.7%)		
*Pregnancy Complications*				
C-section	267 (75.7%)	128 (76.64%)	139 (69.5%)	0.15
Preeclampsia	17 (4.63%)	9 (5.39%)	8 (4.0%)	0.70
FGR	27 (7.35%)	11 (6.58%)	16 (8.0%)	0.75
Preterm Birth	16 (4.36%)	7 (4.19%)	9 (4.50%)	0.99
*Neonatal Characteristics*				
Gender (male)	200 (54.5%)	90 (53.89%)	110 (55.0%)	0.01
GA at birth	39 (1)	39 (1)	39 (1)	0.23
Weight	3340 (520)	3380 (510.0)	3295 (507.5)	0.07
Length	51 (1.5)	51 (2)	50 (1)	0.12
Head Circumference	34 (2)	35 (3)	34 (2)	0.45
APGAR score	9 (0)	9 (1)	9 (0)	0.05

FGR = Fetal Growth Restriction; GA = Gestational Age; *NA* = not applicable.

**Table 2:** Median values of hematological and biochemical tests according to COVID-19 status during pregnancy.

Parameter	Overall	Group 1	Group 2	*p*-Value
*Maternal*				
WBC	9.82 (3.13)	9.69 (2.69)	10.04 (3.19)	0.84
**Platelets**	233 (75)	237.0 (73.0)	229.5 (81.5)	**0.01**
Hemoglobin	11.7 (1.4)	11.6 (1.4)	11.8 (1.4)	0.33
Hematocrit	34.6 (3.7)	34.6 (3.90)	34.5 (3.62)	0.53
Red Blood Cells	3.96 (0.455)	3.97 (0.45)	3.94 (0.45)	0.91
GOT	15.8 (5.45)	15.70 (5.85)	15.95 (5.22)	0.45
GPT	10.4 (5.8)	10.20 (5.25)	10.85 (6.12)	0.25
*Neonatal*				
**WBC**	20.01 (5.86)	20.46 (5.86)	19.69 (6.04)	**0.04**
Platelets	302.00 (74.50)	303 (74.50)	302 (71.75)	0.85
Red Blood Cells	4.54 (0.78)	4.500 (0.70)	4.595 (0.82)	0.40
Hemoglobin	16.30 (2.50)	16.10 (2.55)	16.35 (2.65)	0.22
Hematocrit	47.10 (7.25)	47.0 (6.95)	47.3 (7.37)	0.53
GOT	46.70 (20.60)	48.30 (19.95)	44.85 (19.57)	0.08
GPT	13.90 (7.95)	14.0 (9.65)	13.6 (7.42)	0.09
Unconjugated bilirubine	6.30 (2.45)	6.3 (2.40)	6.2 (2.42)	0.95
Conjugated bilirubine	0.40 (0.20)	0.4 (0.1)	0.4 (0.2)	0.9
Urea	12.50 (6.80)	12.70 (6.20)	12.25 (6.97)	0.26
Creatinine	0.60 (0.10)	0.6 (0.2)	0.5 (0.2)	0.54

**Table 3:** Associations between the COVID-19 condition during pregnancy and the occurrence of specific laboratory abnormalities in newborns.

	Group 1	Group 2	*p*-Value (OR; 95%CI)
Neonatal Anemia	19 (11.4%)	15 (7.5%)	0.21 (1.58; 0.73–3.46)
Neonatal Leukocytosis	9 (5.39%)	6 (3.0%)	0.29 (1.83; 0.57–6.41)
Neonatal Thrombocytopenia	1 (0.59%)	-	*NA*
Elevated GOT level	127 (76.04%)	136 (68%)	0.10 (1.49; 0.91–2.44)
Elevated GPT level	1 (0.59%)	1 (1.0%)	0.99 (0.59; 0.01–11.56)
Elevated Conjugated hyperbilirubinemia	1 (0.59%)	-	*NA*
Elevated creatinine	1 (0.59%)	-	*NA*

*NA* = not applicable.

**Table 4:** Neonatal laboratory outcomes according to the presence of symptoms and maternal hospitalization due to COVID-19.

Outcomes	Asymptomatic COVID-19			Hospitalization during COVID-19		
	*Yes*	*No*	*p*	*Yes*	*No*	*p*
Neonatal Anemia	-	19 (13.2%)	*NA*	2 (22.2%)	17 (10.7%)	0.27
Neonatal Leukocytosis	1 (4.34%)	8 (5.55%)	0.99	-	9 (5.69%)	*NA*
Neonatal Thrombocytopenia	-	1 (0.69%)	*NA*	-	1/0.63%	*NA*
Conjugated Hyperbilirubinemia	-	1 (0.69%)	*NA*	-	1/0.63%	*NA*
Elevated GOT Level	18/78.2%	107/74.3%	0.8	8/88.8%	117/74.0%	0.45
Elevated GPT level	-	1/0.69%	*NA*	-	1/0.63%	*NA*
Elevated creatinine	-	1/0.69%	*NA*	-	1/0.63%	*NA*

*NA* = not applicable.

**Table 5:** Neonatal laboratory outcomes according to the maternal and neonatal COVID-19 immunoreactivity.

Outcomes	Maternal SARS-CoV-2 Immunopositivity			Neonatal SARS-CoV-2 Immunopositivity		
	*Yes*	*No*	*p*	*Yes*	*No*	*p*
Neonatal Anemia	13 (10.7%)	6 (13.3%)	0.59	9/10.3%	10/12.5%	0.80
Neonatal Leukocytosis	5 (4.09%)	4 (8.88%)	0.25	3/3.45	6/7.5%	0.31
Neonatal Thrombocytopenia	1/0.81%	-	*NA*	1/1.1%	-	*NA*
Conjugated Hyperbilirubinemia	-	1/2.2%	*NA*	-	1/1.3%	*NA*
Elevated GOT Level	89/73.0%	36/80.0%	0.42	65/74.7%	60/75.0%	0.96
Elevated GPT level	1/0.81%	-	*NA*	1/1.1%	-	*NA*
Elevated creatinine	-	1/2.2%	*NA*	-	1/1.3%	*NA*

*NA* = not applicable.

## Data Availability

The data sets used and/or analyzed during the present study are available from the first author on reasonable request.

## Appendix A

**Table A1:** List of abbreviations. The abbreviations including in the text are reported alphabetically.

Abbreviation	Full Form
COVID-19	Coronavirus disease
FGR	fetal growth restriction
GA	gestational age
GOT	glutamic oxaloacetic transaminase
GPT	glutamic pyruvic transaminase
GW	gestational weeks
IgG	Immunoglobulin G
IL-1β	interleukin-1β
IL-6	interleukin-6
NICU	neonatal intensive care unit
RBC	red blood cell
WBC	white blood cells
